# Two Weeks of Continuous Opioid Treatment in an Adenine-Induced Mouse Model of Chronic Kidney Disease Exacerbates the Bone Inflammatory State and Increases Osteoclasts

**DOI:** 10.1007/s00223-024-01239-8

**Published:** 2024-06-10

**Authors:** Corinne E. Metzger, Gregory G. Grecco, Landon Y. Tak, Brady K. Atwood, Matthew R. Allen

**Affiliations:** 1 Department of Anatomy, Cell Biology, and Physiology, Indiana University School of Medicine, 635 Barnhill Drive, MS 5045, Indianapolis, IN 46202 USA; 2 Department of Pharmacology & Toxicology, Indiana University School of Medicine, Indianapolis, IN 46202 USA; 3 Stark Neurosciences Research Institute, Indiana University School of Medicine, Indianapolis, IN 46202 USA; 4 Roudebush Veterans Administration Medical Center, Indianapolis, IN 46202 USA

**Keywords:** Chronic kidney disease, Opioids, Oxycodone, Osteoclasts, Inflammation

## Abstract

Patients with chronic kidney disease (CKD) report high pain levels, but reduced renal clearance eliminates many analgesic options; therefore, 30–50% of CKD patients have chronic opioid prescriptions. Opioid use in CKD is associated with higher fracture rates. Opioids may directly alter bone turnover directly through effects on bone cells and indirectly via increasing inflammation. We hypothesized that continuous opioid exposure would exacerbate the high bone turnover state of CKD and be associated with elevated measures of inflammation. Male C57Bl/6J mice after 8 weeks of adenine-induced CKD (AD) and non-AD controls (CON) had 14-day osmotic pumps (0.25-µL/hr release) containing either saline or 50-mg/mL oxycodone (OXY) surgically implanted in the subscapular region. After 2 weeks, all AD mice had elevated blood urea nitrogen, parathyroid hormone, and serum markers of bone turnover compared to controls with no effect of OXY. Immunohistochemical staining of the distal femur showed increased numbers of osteocytes positive for the mu opioid and for toll-like receptor 4 (TLR4) due to OXY. Osteocyte protein expression of tumor necrosis factor-α (TNF-α) and RANKL were higher due to both AD and OXY so that AD + OXY mice had the highest values. Trabecular osteoclast-covered surfaces were also significantly higher due to both AD and OXY, resulting in AD + OXY mice having 4.5-fold higher osteoclast-covered surfaces than untreated CON. These data demonstrate that opioids are associated with a pro-inflammatory state in osteocytes which increases the pro-resorptive state of CKD.

**Supplementary Information:**

The online version contains supplementary material available at 10.1007/s00223-024-01239-8.

## Introduction

Chronic kidney disease (CKD) is associated with loss of bone and increased fracture rates [1–3]. Complications following fractures are also higher in patients with CKD leading to greater rates of hospitalization and increased post-fracture mortality [2, 4]. Multiple factors increase the risk of fractures in CKD patients including age, high circulating levels of parathyroid hormone, and various classes of drugs [1]. Opioid pain medications increase fracture risk in CKD patients with approximately a twofold higher relative risk of new fracture for CKD patients on these medications [1]. CKD patients with chronic opioid prescriptions have higher rates of hip fractures [5] with a dose-dependent response with higher doses associated with higher hip fractures [6]. Therefore, CKD-induced bone loss compounded with opioid use has greater deleterious effects on skeletal fragility than CKD alone.

Chronic pain is a complication of CKD reported in approximately 70% of patients [7–9], nearly three times higher than the non-CKD population [10]. Over 90% of patients with end-stage renal disease (ESRD) report chronic pain [11]. Due to complications with reduced renal clearance in CKD, there are few pharmaceutical treatment options for pain management leading to 30–50% of CKD patients having at least one opioid prescription each year [9]. Rates are even higher in patients with ESRD [12, 13]. The use of opioids as an analgesic in chronic non-cancer pain is limited whenever possible; however, patients with CKD represent a unique and growing population where, without other suitable and safe alternatives, opioids remain a primary analgesic option.

In the general population, opioid use has been associated with increased fracture risk [14–16]. In older non-CKD patients on opioids for chronic pain, opioids lead to a dose-dependent increase in fracture risk [17]. This effect is independent of an opioid effect on falls [18] suggesting a direct effect of opioids on bone fragility. In non-CKD bone, opioids have been shown to decrease bone formation rate and reduce trabecular bone volume [19]. In an in vitro study, osteoblast-like cells expressed all three types of opioid receptors [20]; however, how this corresponds to in vivo bone cells is not clear. Another study found that osteoblast activity in cultured osteoblasts from human trabecular bone is inhibited by opioids, both endogenous and exogenous [21]. Opioids may also indirectly impact bone by increasing inflammation, as opioids bind to an accessory protein on toll-like receptor 4 (TLR4) activating pro-inflammatory signaling [22, 23]. In the setting of CKD with secondary hyperparathyroidism, where osteoclastic drive is already high, opioid-induced exacerbation of resorption could heighten the degree and rate of bone loss and skeletal fragility.

In the current study, we aimed to assess the impact of 2 weeks of continuous opioid treatment on bone in mice with established adenine-induced CKD. Oxycodone was selected for treatment as it is a commonly prescribed opioid analgesic and considered a safe analgesic option with appropriate dosing in CKD patients [24]. We hypothesized that oxycodone treatment would lead to greater bone inflammatory cytokines and osteoclasts compared to non-opioid-treated mice and that this effect would be exacerbated in adenine-induced CKD.

## Methods

**Fig. 1:**
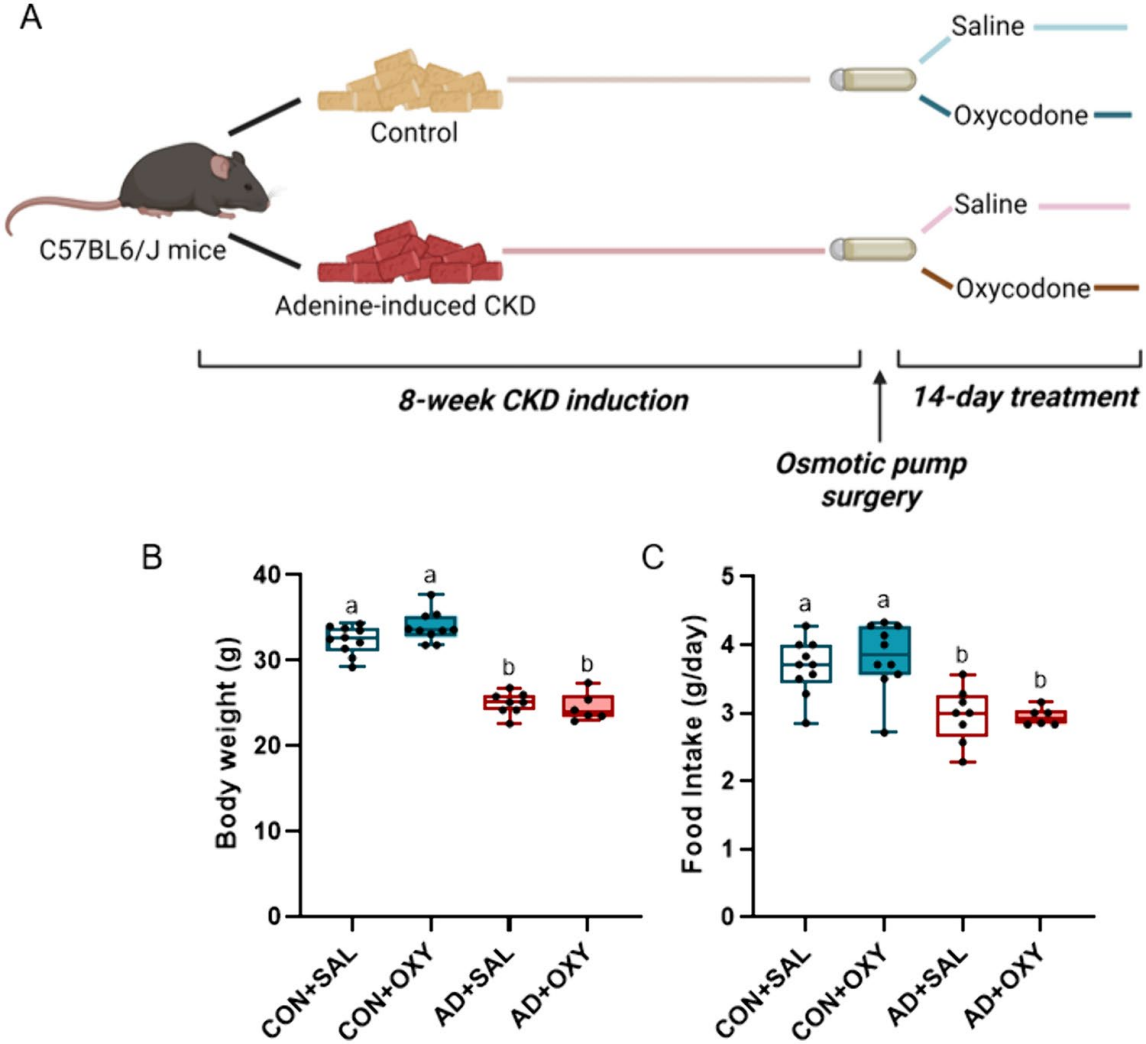
Timeline of study and body weight and food intake at study endpoint. **A** Schematic of study timeline. Figure created with BioRender. **B** Control mice weighed more than adenine mice with no effect of oxycodone treatment. **C** Food intake was lower in adenine mice compared to control mice regardless of oxycodone treatment. Bars not sharing the same letter are statistically different (*p* < 0.05)

*Animals and study timeline:* Male C57Bl/6 J mice (JAX #000664; *n* = 40) were ordered from Jackson Laboratories (Bar Harbor, ME, USA) at 15 weeks of age and group housed 3–5 per cage at an institutionally approved animal facility with 12-h light/dark cycles. For the current study, only male mice were used as CKD impacts bone in both male and female mice [25], continuous opioid exposure had a greater effect on bone in male mice [19], and the larger body size of male mice vs. female would better handle the 2-week osmotic pump. At 16 weeks of age, all control mice (CON; *n* = 20) were switched to a purified casein-based diet with 0.9% phosphorous and 0.6% calcium (Teklad Diets [TD.150303]; Inotiv, Madison, WI, USA). All adenine-CKD mice (AD; *n* = 20) were switched to an identical casein-based diet with the inclusion of 0.2% adenine (Teklad Diets [TD.170948]; Inotiv). After a 6-week induction on the adenine diet, all AD mice were switched back to the control casein-based diet for the remaining four weeks of the study [25, 26]. CON groups remained on the casein-based diet without adenine for the entire study. After eight weeks, all mice had a 14-day osmotic pump with 0.25-µL/hr release rate surgically implanted in the scapular region (ALZET Osmotic Pumps, Cupertino, CA, USA). Briefly, mice were anesthetized with inhaled vaporized isoflurane (~ 1.5%) and kept warm on an infrared heating pad. The scapular region was shaved and cleaned with iodine and 70% ethanol. A single incision was made in the upper back between the scapula and the filled osmotic pump was placed with the port side facing caudally. Tissue adhesive was used to close the incision. A single dose of carprofen (5 mg/kg, s.c.) was given while under anesthesia and lidocaine was topically placed on the incision after the tissue adhesive dried. Osmotic pumps filled with 50-mg/mL oxycodone (OXY) were placed in *n* = 10 mice of both CON and AD mice (Fig. 1A). All remaining AD and CON mice received pumps filled with sterile saline (SAL). All mice were then allowed to recover from anesthesia in a cage free from bedding with a heating source at one side. After several hours of monitoring, mice were returned to cages with normal bedding and nesting material and housed singly for the remainder of the study. Three AD mice (one with saline and two with oxycodone pumps) were euthanized within 48 h of surgery due to hunched appearance and dehydration that did not respond to supportive measures. Food intake was measured in the second week following the surgery. Two weeks after pump implantation, all animals were anesthetized via inhaled isoflurane and euthanized. All animal procedures were approved by the Indiana University School of Medicine Animal Use and Care Committee prior to the initiation of experimental protocols and methods were carried out in accordance with relevant guidelines and regulations.

*Serum biochemistries*: Cardiac blood collected at time of euthanasia was used to measure serum blood urea nitrogen (BUN) via colorimetric assay to assess the presence of kidney disease (BioAssay Systems, Hayward, CA, USA). Serum 1–84 parathyroid hormone (PTH) was measured via ELISA (Immnotopics Quidel, San Diego, CA, USA). Serum N-terminal propeptide of type 1 procollagen (PINP) was measured with EIA (Immunodiagnostic Systems, East Bolden, United Kingdom) and serum TRAcP 5b was also measured via EIA (Immunodiagnostic Systems). All serum assays were run in duplicate following manufacturer procedures.

*Micro-computed tomography*: The right distal 1/3 femora were scanned 3 at a time on a SkyScan 1172 system (Bruker, Billerica, MA, USA) with a 0.5 aluminum filter and a 8-µm voxel size. Trabecular bone was analyzed in a 1-mm region starting proximal to the growth plate in the distal femur. Cortical bone properties were analyzed on 5 contiguous slices located ~ 2.5 mm proximal to growth plate in the distal femur. Cortical porosity was assessed from hand-drawn regions of interest on five contiguous slices, tracing the periosteal and endosteal surfaces. Cortical porosity was defined as the inverse of bone volume or the percent of void space within the cortical bone region (i.e., 95% bone volume = 5% cortical porosity). A single average value from across the five slices was obtained.

*Immunohistochemistry*: After scanning, the right distal femur was decalcified in 14% EDTA for ~ 2 weeks. Samples were subsequently embedded in paraffin and sectioned at 5 µm thickness. Sections were stained utilizing a standard avidin–biotin method. Samples were stained for the mu opioid receptor (ImmunoStar, Inc., Hudson, WI, USA), toll-like receptor 4 (TLR4; Abcam, Cambridge, MA, USA), tumor necrosis factor-α (TNF-α; Abcam), and receptor activator of nuclear facto κB ligand (RANKL; Abcam). Peroxidase development was performed with an enzyme substrate kit (DAB, Vector Laboratories, Burlingame, CA, USA) and counterstaining was conducted with methyl green (Vector Laboratories) which stain osteocyte nuclei as previously described [26]. Negative controls for all antibodies were completed by omitting the primary antibody. Analyses were completed in both the trabecular bone excluding primary spongiosa and endocortical surfaces and in the cortical bone closest to midshaft. Results are reported as the percentage of osteocytes stained positively for the protein (DAB-positive) relative to all osteocytes (DAB positive and methyl green positive) in the regions of interest. All analyses were completed by the same individual.

*TRAP staining for osteoclast-covered surfaces*: For osteoclast-covered surfaces, 5-µm paraffin-embedded sections of the right distal femur were stained with tartrate resistant alkaline phosphatase (TRAP) with toluidine blue as a counterstain. Samples were analyzed as osteoclast-covered trabecular surfaces normalized to total trabecular bone surface (Oc.S/BS, %) and osteoclast number to bone perimeter (Oc.N/B.Pm, #/mm) in a standard region of interest of trabecular bone excluding primary spongiosa and endocortical surfaces near the distal growth plate. Analyses were performed using BIOQUANT (BIOQUANT Image Analysis, Nashville, TN).

*Histological osteoid assessment*: Fixed undemineralized left distal femurs were serially dehydrated through graded steps of ethanol and subsequently embedded in methyl methacrylate (Sigma Aldrich, St. Louis, MO). Serial frontal sections were cut 4 µm thick and stained with Von Kossa/McNeal stain for assessment of trabecular osteoid surfaces (OS/BS, %) and osteoid thickness (O.Th, µm). These measurements were taken utilizing a similar trabecular region of interest as described above for TRAP stain analysis. All nomenclature for histomorphometry follows standard usage [27].

*Statistical analyses*: All data were tested for normality with Levene’s test for equality of variances. If data met assumptions of normality, a 2 × 2 ANOVA (adenine by oxycodone) was completed with main and interaction effects recorded. Effect sizes (ES; partial *η*^2^) were recorded for statistically significant main and interaction effects. If the model ANOVA was statistically significant (*p* < 0.05), a Tukey post hoc was completed. If data did not meet normality, a Kruskal–Wallis test was completed. If the model *p*-value was statistically significant, pairwise comparisons were completed with Bonferroni correction for multiple tests. Regression analyses were performed with PTH as the independent variable and trabecular osteocyte TNF-α, RANKL, TLR4, and osteoclast-covered trabecular surfaces independently. Secondly, osteocyte TNF-α and TLR4 were independent variables to osteocyte RANKL. Finally, osteoclast-covered trabecular surfaces were assessed as the dependent variable against osteocyte TNF-α, RANKL, and TLR4 independently. All statistical analyses were completed with IBM SPSS Statistics 28 (IBM, Armonk, NY, United States). All data are represented as mean ± standard deviation showing individual data points.

## Results

*Body weight and food intake*: At study endpoint, there was a main effect of AD on body weight (*p* < 0.001, ES = 0.874) with no effect of OXY (*p* = 0.570) or an interaction (*p* = 0.178). Both CON groups had higher body weight than both AD groups regardless of OXY treatment (Fig. 1B). Similar trends were seen with food intake with a main effect of AD (*p* < 0.001, ES = 0.501), but no effects of OXY (*p* = 0.942) nor an interaction effect (*p* = 0.836). Both CON mice had higher food intake in the second full week post-surgery compared to both AD groups (Fig. 1C).

**Fig. 2:**
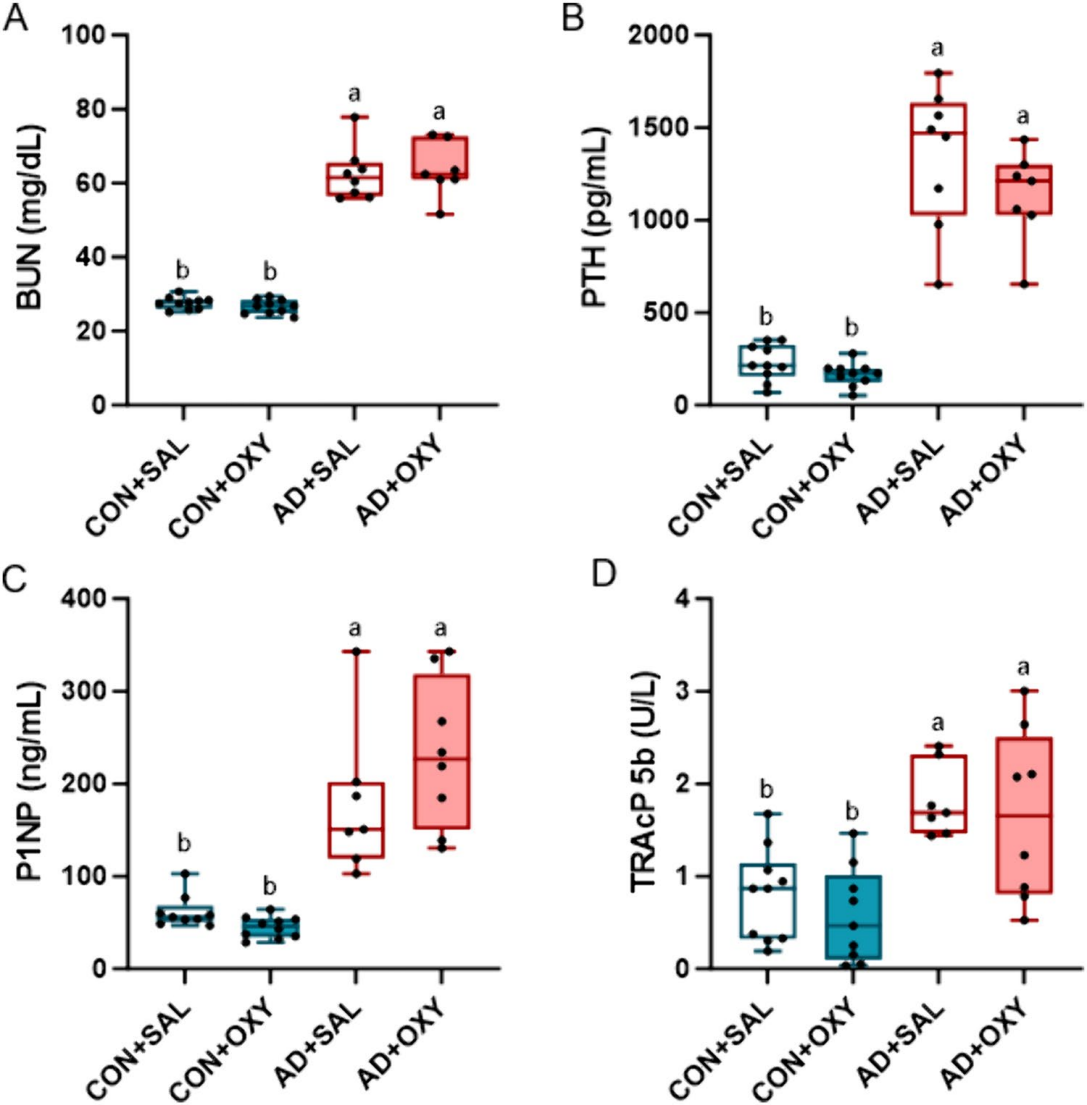
Serum markers of kidney disease and bone turnover. **A** Serum BUN was higher on both AD groups compared to CON groups. **B** Serum PTH was elevated in AD groups compared to CON groups. **C** Serum P1NP was higher in AD vs. CON groups. **D** Serum TRAcP 5b was elevated in both AD groups vs. CON groups. Bars not sharing the same letter are statistically different (*p* < 0.05)

*Serum measures*: All serum measures did not meet normality assumptions and were run with a Kruskal–Wallis test. For BUN (test statistic = 25.488, *p* < 0.0001), both AD groups were higher compared to both CON groups (Fig. 2A). Likewise, PTH (test statistic = 26.406, *p* < 0.0001), P1NP (test statistic = 26.161, *p* < 0.001), and TRAcP 5b (test statistic = 15.831, *p* = 0.001) all showed statistical difference with AD groups higher than CON groups (Fig. 2B–D).

**Fig. 3:**
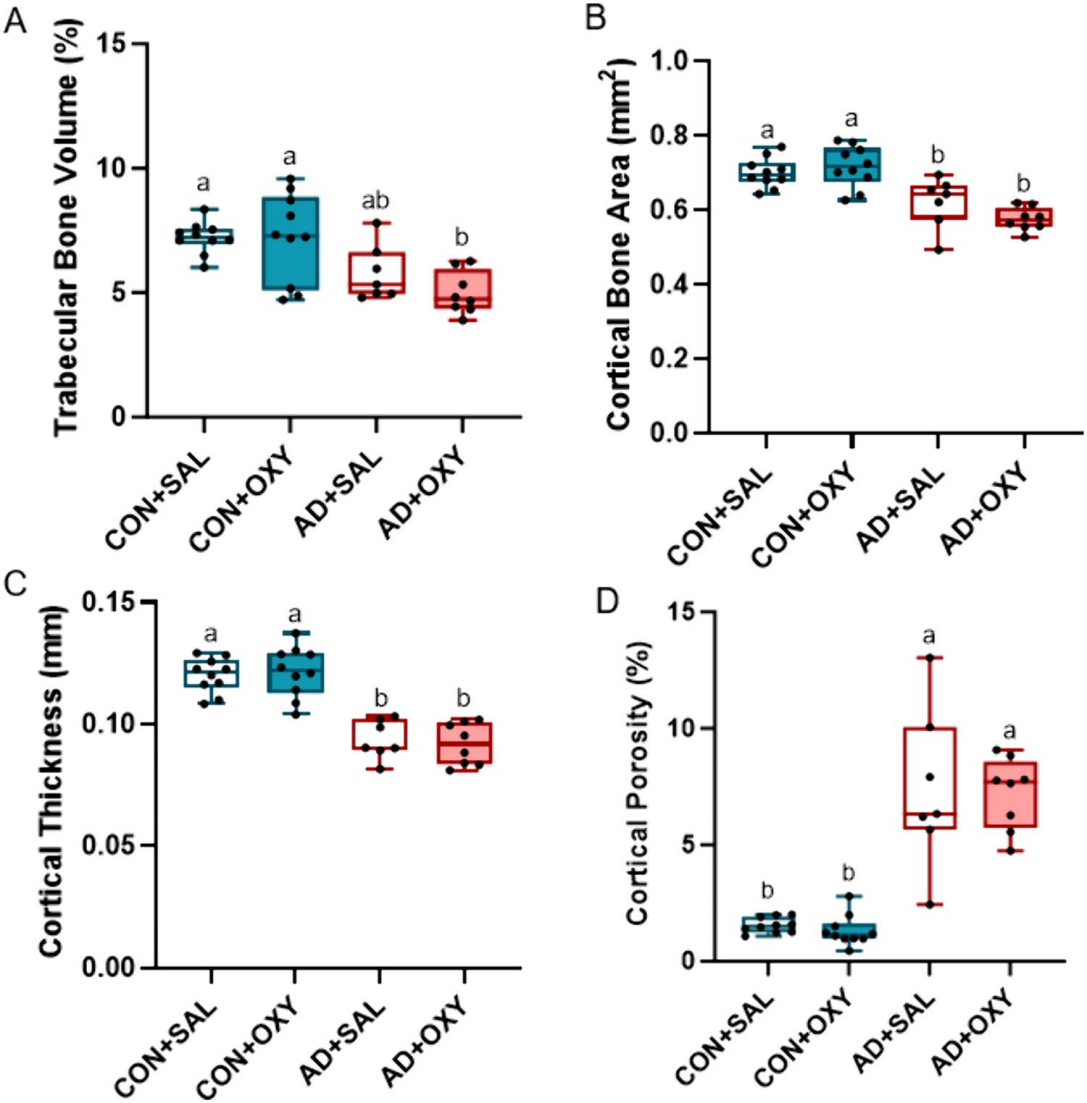
Femoral microarchitecture parameters. **A** Trabecular bone volume in AD + OXY mice was lower than both CON groups. **B** Cortical bone area was lower in both AD groups vs. both CON groups. **C** Cortical thickness was lower in AD vs. CON regardless of treatment. **D** Cortical porosity was higher in both AD groups compared to both CON groups. Bars not sharing the same letter are statistically different (*p* < 0.05)

*Bone microarchitecture*: For trabecular bone volume, there was an effect of AD (*p* < 0.001, ES = 0.390), but no effect of OXY (*p* = 0.340) nor an interaction (*p* = 0.340). AD + OXY was lower than both CON groups. The untreated AD group was not different from either CON group or the AD + OXY group (Fig. 3A). Other trabecular parameters also showed main effects of AD (trabecular thickness *p* < 0.013, trabecular separation *p* < 0.001, trabecular number *p* < 0.001), but no effect of OXY and no interaction effects (Table 1).

**Table 1:** Distal femur trabecular bone parameters from micro-CT—trabecular thickness (Tb.Th), trabecular separation (Tb.Sp), and trabecular number (Tb.N). Groups not sharing the same letter are statistically different from each other (*p* < 0.05)

	**Tb.Th (mm)**	**Tb.Sp (mm)**	**Tb.N (#/mm)**
*CON* + *SAL*	0.0390 ± 0.001^b^	0.2456 ± 0.016^b^	1.8549 ± 0.174^a^
*CON* + *OXY*	0.0391 ± 0.002^b^	0.2611 ± 0.022^b^	1.8279 ± 0.371^a^
*AD* + *SAL*	0.0426 ± 0.003^a^	0.3158 ± 0.016^a^	1.3482 ± 0.156^b^
*AD* + *OXY*	0.0401 ± 0.003^ab^	0.3075 ± 0.012^a^	1.2386 ± 0.168^b^

**Fig. 4:**
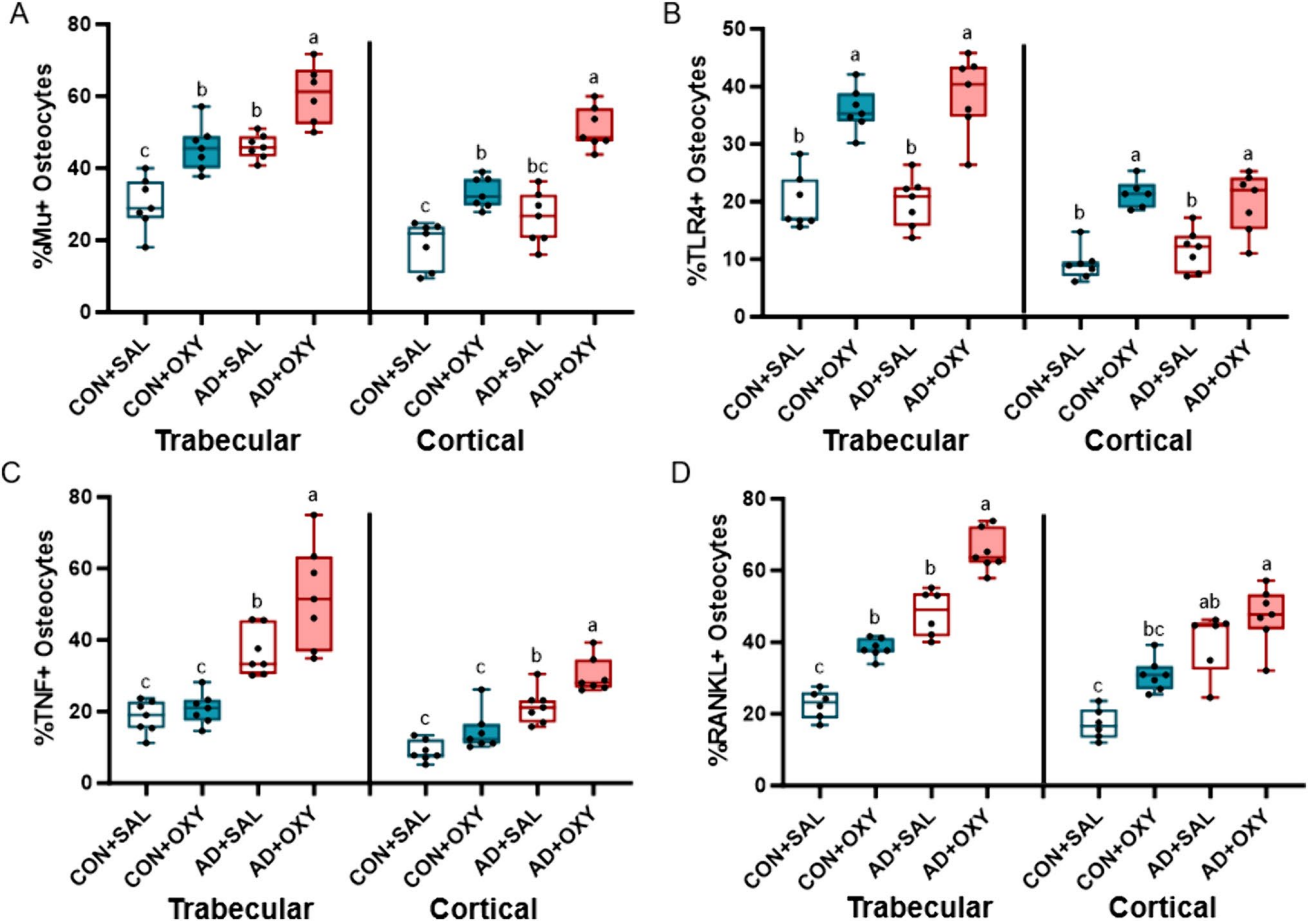
Immunohistochemical analyses on trabecular and cortical bone of the distal femur. **A** Mu receptor-positive osteocytes were elevated due to both adenine and oxycodone treatment with AD + OXY having the highest levels of all groups. **B** TLR4-positive osteocytes were higher in both cortical and trabecular bone due to oxycodone treatment with no elevation due to adenine. **C** TNF-α-positive osteocytes were higher in AD + SAL mice vs. both CON groups with AD + OXY being higher than AD + SAL. **D** RANKL-positive osteocytes were elevated due to both adenine and oxycodone with AD + OXY having the highest value of all groups. Bars not sharing the same letter are statistically different (*p* < 0.05)

There were effects of AD for both cortical bone area (*p* < 0.001, ES = 0.576; Fig. 3B) and cortical thickness (*p* < 0.001, ES = 0.744; Fig. 4C), but no effects of OXY nor interaction effects. For both, CON groups had higher values than AD groups. Cortical porosity did not meet normality assumptions, but the Kruskal–Wallis test showed statistical difference (*p* < 0.001). Cortical porosity was higher in AD groups compared to CON groups with no differences between SAL and OXY groups (Fig. 3D).

*Immunohistochemical analyses*: For mu opioid receptor-positive osteocytes in trabecular bone, there were main effects of AD (*p* < 0.001, ES = 0.617) and OXY (*p* < 0.001, ES = 0.609), but no interaction effect (*p* = 0.884). The AD + OXY group had the highest mu opioid receptor-positive osteocytes followed by AD + SAL and CON + OXY with CON + SAL having the lowest group value (Fig. 4A). In the cortical shaft, there were main effects of AD (*p* < 0.001, ES = 0.556) and OXY (*p* < 0.001, ES = 0.755) and an interaction effect (*p* = 0.029, ES = 0.184). AD + OXY was highest followed by CON + OXY. The CON + SAL group had the lowest group average (Fig. 4A; Supplemental Figure). For TLR4-positive osteocytes in trabecular bone, there was only a main effect of OXY (*p* < 0.001, ES = 0.777) and no effect of AD (*p* = 0.492) nor an interaction effect (*p* = 0.507). Both OXY groups had higher TLR4-positive osteocytes compared to both SAL groups (Fig. 4B). In the cortical shaft, there was also only a main effect of OXY (*p* < 0.001, ES = 0.687) and not an AD (*p* = 0.751) or interaction effect (*p* = 0.185). In the cortical shaft, both OXY groups were higher than both SAL groups (Fig. 4B). For TNF-α-positive osteocytes in the trabecular bone, there were effects of AD (*p* < 0.001, ES = 0.709) and OXY (*p* = 0.001, ES = 0.245), but no interaction (*p* = 0.050). AD + OXY had the highest TNF-α-positive osteocytes followed by AD + SAL. Both CON groups had the lowest values (Fig. 4C). Within cortical bone, there were also effects of AD (*p* < 0.001, ES = 0.725) and OXY (*p* = 0.001, ES = 0.398) with no interaction effect (*p* = 0.395). Like the trabecular bone, AD + OXY was highest followed by AD + SAL with both CON groups having the lowest values (Fig. 4C). For RANKL-positive osteocytes in trabecular bone, there was a main effect of AD (*p* < 0.001, ES = 0.868) and OXY (*p* < 0.001, ES = 0.759), but no interaction effect (*p* = 0.461). AD + OXY was higher than AD + SAL and CON + OXY with CON + SAL having the lowest group value (Fig. 4D). In the cortical bone, there were main effects of AD (*p* < 0.001, ES = 0.625) and OXY (*p* < 0.001, ES = 0.422) with no interaction effect (*p* = 0.307). AD + OXY mice had the highest value with CON + SAL mice having the lowest group value (Fig. 4D).

**Fig. 5:**
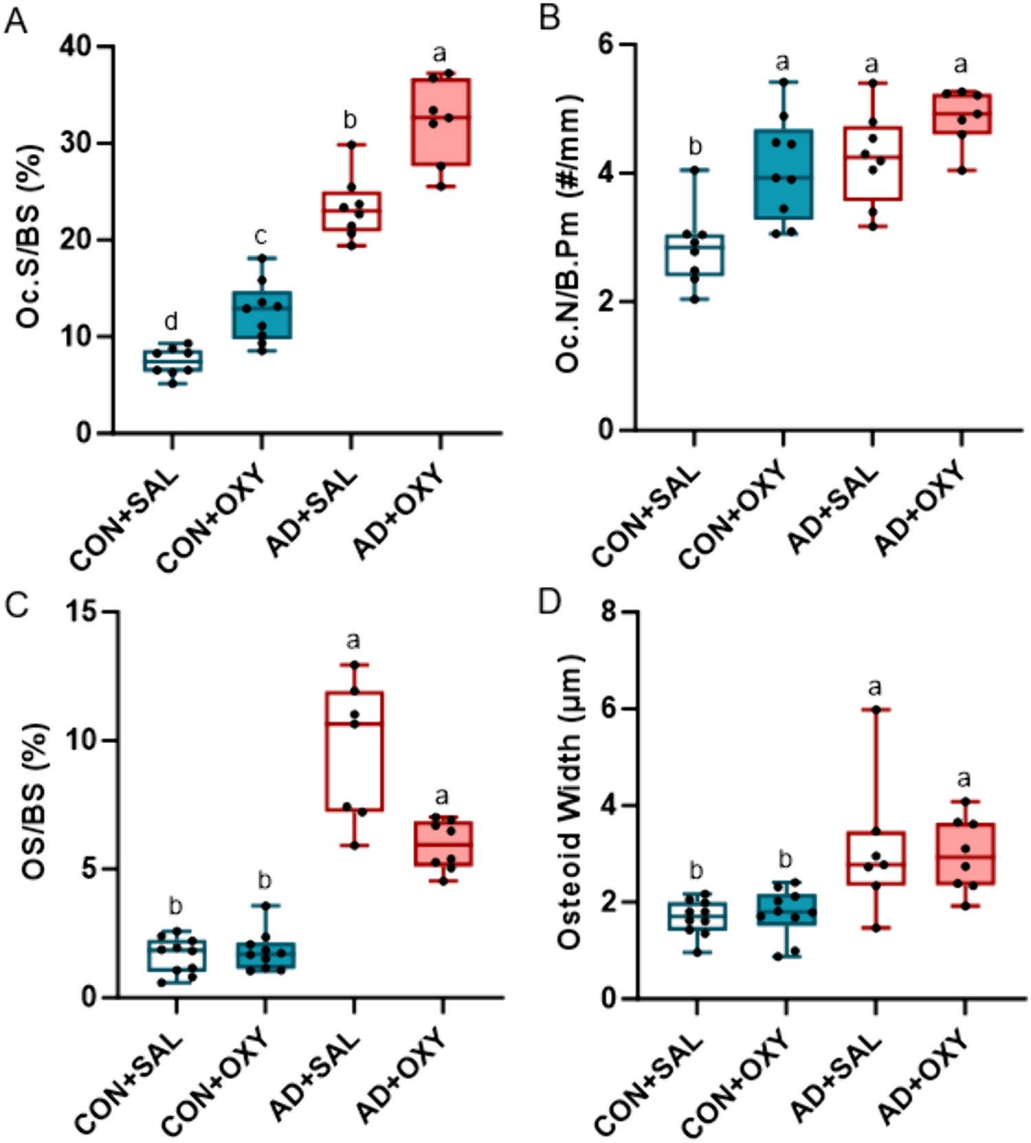
Histological assessment of osteoclasts and osteoid in trabecular bone of the distal femur. **A** Osteoclast-covered trabecular surfaces (Oc.S/BS) were lowest in CON + SAL with increases due to both oxycodone and adenine with AD + OXY having the highest group value. **B** Osteoclast numbers (Oc.N/B.Pm) were lower in CON + SAL vs. all other groups. **C** Osteoid-covered trabecular surfaces (OS/BS) were higher in both adenine groups compared to both control groups. **D** Osteoid width was elevated in both adenine groups compared to control groups with no effect of oxycodone treatment. Bars not sharing the same letter are statistically different (*p* < 0.05)

*Trabecular osteoclast surfaces and numbers*: For osteoclast-covered trabecular surfaces, there were effects of both AD (*p* < 0.001, ES = 0.908) and OXY (*p* < 0.001, ES = 0.618) with no interaction effect (*p* = 0.055). OcS/BS was highest in the AD + OXY group followed by AD + SAL, then CON + OXY with the CON + SAL group lowest of all groups (Fig. 5A). For osteoclast numbers, there was a main effect of AD (*p* < 0.001, ES = 0.431) and OXY (*p* < 0.001, ES = 0.356), but no interaction effect (*p* = 0.224). CON + SAL had the lowest number of osteoclasts on bone perimeter across all the groups (Fig. 5B).

*Trabecular osteoid surfaces and width*: Osteoid-covered trabecular surfaces failed normality assumptions and a Kruskal–Wallis was utilized (test statistic = 26.471, *p* < 0.001). Both AD groups were higher than CON groups (Fig. 5C). Osteoid width showed only an effect of AD (*p* < 0.001, ES = 0.432) with no OXY (*p* = 0.961) or interaction effect (*p* = 0.695). Osteoid width was higher in AD compared to CON groups (Fig. 5D).

*Regression analyses*: Regression analyses demonstrated a significant relationship between circulating PTH and osteocyte TNF-α (*R*^2^ = 0.470, *p* < 0.0001) and PTH and osteocyte RANKL (*R*^2^ = 0.274, *p* = 0.004), but no relationship between PTH and TLR4 (*R*^2^ = 0.004, *p* = 0.303). When assessing regression with osteocyte RANKL as the dependent variable, both TNF-α (*R*^2^ = 0.533, *p* < 0.001) and TLR4 (*R*^2^ = 0.238, *p* = 0.007) independently statistically accounted for some of the variability in RANKL. When assessing regression analyses with osteoclast-covered trabecular surfaces as the dependent variable, serum PTH (*R*^2^ = 0.443, *p* < 0.0001), osteocyte TNF-α (*R*^2^ = 0.677, *p* < 0.0001), and osteocyte RANKL (*R*^2^ = 0.792, *p* < 0.0001) all independently accounted for the variability in osteoclast-covered trabecular surfaces. Osteocyte TLR4 did not reach a statistically significant regression with osteoclast-covered trabecular surfaces (*R*^2^ = 0.095, *p* = 0.069).

## Discussion

The primary finding of this study is that two weeks of continuous opioid exposure in mice with adenine-induced CKD leads to an exacerbated inflammatory state in osteocytes and increased osteoclasts. Without altering indices of kidney function or circulating PTH levels, two weeks of continuous oxycodone treatment resulted in a greater percentage of osteocytes positive for both the mu opioid receptor and TLR4. Both adenine-induced CKD and oxycodone treatment led to greater levels of osteocytes positive for RANKL resulting in opioid-treated CKD mice having the highest values of all. Overall, two weeks of short-term exposure to oxycodone led to elevated osteoclasts which, when combined with the pro-osteoclast setting of CKD could eventually lead to detrimental effects on bone.

As opioids have been widely used and misused over many years, epidemiological studies demonstrate connections to reduced bone mass[28–33] and elevated fracture risk with long-term use [34]. For example, individuals with an opioid use disorder are 4.13 × more likely to fracture than those without [35]. Meta-analyses have reported ~ 1.88 relative risk of any fracture in patients with chronic opioid use for non-cancer pain [15] and an approximate 1.54 relative risk of specifically hip fractures with opioid use [14]. Additionally, studies have shown increased fracture risk in older adults with opioid treatment [18, 36, 37]. Importantly, the association between opioid use and fractures is stronger than opioid use and falls/fall injuries [18] indicating that the increased fracture rate in older adults is not solely due to an increase in falls. In CKD where fracture rates are already higher than an age-matched population [2, 3, 38, 39], the compounding influence of opioids on bone mass and bone fragility require exploration. Two studies have shown increased fracture rates in CKD patients with both short- and long-term use of opioids [5] as well as an opioid dose-dependent increase in fractures [6]. Therefore, epidemiological studies demonstrate increased bone loss and elevated fracture risk with long-duration opioid use.

In the clinical setting, oxycodone is considered a moderately safe opioid to use for chronic analgesia in CKD when dosed properly [24]; therefore, in our current study, we aimed to assess oxycodone treatment in mice with established adenine-induced CKD. All CKD mice in this study had elevated serum BUN indicating the presence of kidney disease as well as high PTH which matches our previous work with this model of CKD [25]. Two weeks of oxycodone treatment had no effect on BUN nor PTH in our current study. Additionally, the oxycodone treatment also had no impact on body weight, food intake, or overall animal appearance/behavior.

CKD with secondary hyperparathyroidism is associated with high bone turnover in both human [40] and animal models [25] and in the current study, both P1NP and TRAcP 5b, serum markers of bone turnover, were elevated in all adenine mice. In vitro studies have demonstrated an inhibitory effect of endogenous opioids (met-enkephalin) on osteoblasts and this inhibitory effect is abolished with the addition of the naltrexone, a competitive antagonist of opioid receptors [21]. Similarly, mice lacking dynorphin, an endogenous opioid that binds to the kappa opioid receptor, had higher bone mass and bone turnover than wild-type counterparts [41]. Therefore, opioids may have a direct effect on bone cells, particularly an inhibitory effect on osteoblasts. We were unable to directly measure bone formation rate with dynamic histomorphometry in the current study due in part to the shorter time of treatment. Our systemic marker of formation was not altered due to oxycodone treatment, but our 2-week period of exposure could have been too short to influence systemic markers of formation. For example, sustained morphine delivery for 25 days in healthy male mice reduced bone formation rate [19], while our study only had 14 days of treatment. In the current study, we measured trabecular osteoid and found that both osteoid-covered trabecular surfaces and osteoid width were elevated in our CKD mice. While there was not a statistical difference in osteoid-covered trabecular surfaces, likely due to the high variability in the adenine group, the oxycodone-treated CKD group had ~ 40% lower osteoid-covered trabecular surfaces than the saline-treated adenine group which may indicate a mild suppression of bone forming in these mice. Future work should include studies with fluorochrome labels to be able to directly assess bone formation rate via dynamic histomorphometry.

After the 2-week period of treatment, the oxycodone-treated mice in our study showed different mu opioid receptors presence in the osteocytes. Previous work has established that cultured osteoblast-like cells express opioid receptors [20]. To our knowledge, no other studies have shown mu opioid receptor expression in mouse osteoblasts or osteocytes; however, the antibody utilized is well-reported with specificity to the receptor. In our current study, we found osteocytes, terminally differentiated cells from the osteoblast lineage, positively stained for the mu opioid receptor and the percentage of osteocytes that stained positively was ~ 30% higher in the oxycodone-treated mice. Therefore, from our immunohistochemical analysis, it appears that continuous opioid treatment increases mu receptor protein expression. Interestingly, there were also main effects of adenine on the mu receptor in both trabecular and cortical bone indicating the disease process itself may alter endogenous opioid activity or responses. Further work needs to be done to validate the presence and role of the mu opioid receptor in bone cells in various species.

Beyond direct effects of opioids acting through opioid receptors, evidence points to indirect effects of opioids leading to increased inflammation. CKD alone is characterized by a chronic pro-inflammatory state and inflammatory factors in CKD are associated with overall mortality [42, 43] and TLR4 signaling is induced by the uremic environment [44]. Apart from CKD, opioid receptor agonists also activate the TLR4 pathway by binding to an accessory protein on TLR4 [22]. This, in turn, could activate pro-inflammatory signaling, upregulate NF-κB expression, and increase overall pro-inflammatory cytokines [23]. In the condition of CKD, this increase in inflammatory drive could exacerbate CKD-induced inflammation. In our study, osteocytes positive for TLR4 were not different due to only CKD; however, TLR4-positive osteocytes were nearly twofold higher in oxycodone-treated mice regardless of CKD status. Importantly, circulating PTH was not associated with TLR4-positive osteocytes (R^2^ = 0.004) indicating the alterations were likely primarily driven by opioid exposure. The elevated TLR4 may have a compounding effect on TNF-α-positive osteocytes in CKD mice as levels were 44% higher in oxycodone-treated adenine mice vs. untreated adenine mice, while oxycodone treatment only caused an 11% difference between the non-adenine groups. Additionally, protein expression of RANKL, a downstream target of the NF-κB signaling pathway activated by TLR4, was higher due to both CKD and oxycodone treatment resulting in nearly ~ 2.7-fold higher osteocyte RANKL in adenine–oxycodone-treated mice vs. control saline-treated mice. Regression analyses showed that both TLR4-positive osteocytes and TNF-positive osteocytes were significant predictors of the variability in RANKL-positive osteocytes; however, TLR4 alone was not a predictor of osteoclast-covered surfaces. Therefore, in our model of continuous oxycodone treatment, oxycodone alone increased TLR4, but the combination with adenine-induced CKD led to higher TNF-α and RANKL indicating a greater pro-inflammatory state than CKD alone.

A key hallmark of the bone phenotype of CKD with high PTH is rampant osteoclastic resorption [25, 26] which leads to bone loss and cortical bone resorption in the form of cortical porosity. After only two weeks of oxycodone treatment in mice with established CKD, there were not notable differences in bone structural parameters beyond expected changes due to CKD except for trabecular bone volume a 14% lower in AD + OXY than untreated adenine, but only statistically lower than both CON groups. Cortical bone area was 8% lower in oxycodone-treated adenine mice compared to untreated adenine mice, but this did not approach statistical difference. These changes allude to potential resorption-driven bone loss with oxycodone treatment which could become more apparent with longer duration of treatment. Oxycodone treatment did result in 40–70% higher osteoclast-covered trabecular surfaces compared to matched controls. Due to both CKD-induced increases in osteoclasts and oxycodone-induced increases in osteoclasts, adenine mice treated with oxycodone had 4.5-fold  higher osteoclast-covered surfaces than the saline control group. Exacerbating the already high osteoclastic drive of CKD with chronic opioids could lead to even greater bone loss over time than CKD alone.

A limitation of the current study is that we only assessed male mice. We previously have shown similar phenotypes in male and female C57Bl/6 mice with adenine-induced CKD [25], but the impact of opioids in the context of CKD may vary between sexes. In humans, the impact of long-term opioid exposure on bone appears to be stronger in males vs. females [30, 33, 45]. Additionally, 25 days of sustained morphine administration in mice impacted the bone of male mice, but not female mice [19]. Therefore, future studies should assess opioids in the context of the CKD-induced bone phenotype in both males and females. As previously mentioned, we were unable to directly measure bone formation rate via dynamic histomorphometry and, therefore, cannot directly assess tissue-specific changes in bone formation rate. These measures would add valuable information about bone-specific changes in future studies. Furthermore, our study assessed only a 2-week chronic exposure to oxycodone and longer durations will need to be assessed in future studies which would also allow for better assessment of tissue-specific changes in bone formation rate.

Overall, this study demonstrates that two weeks of continuous oxycodone treatment in mice with biochemical and skeletal manifestations consistent with CKD resulted in an elevated pro-inflammatory state within bone and increased osteoclasts. With prolonged treatment, these changes could lead to significantly greater bone loss and skeletal fragility than CKD alone. These data allude to the importance of understanding the mechanisms of opioid-induced bone changes as well as how those changes interact with different disease states like CKD. Additionally, these data highlight the need for the development of safer, alternative analgesic options for CKD patients with high pain burden.

### Supplementary Information

Below is the link to the electronic supplementary material.

Supplementary file1 (DOCX 333 KB)

## Declarations

### Research Involving Human and Animal Participants

All animal procedures were approved by the Indiana University School of Medicine Animal Use and Care Committee prior to the initiation of experimental protocols and methods were carried out in accordance with relevant guidelines and regulations.

## References

[1] Jadoul M, Albert JM, Akiba T (2006). Incidence and risk factors for hip or other bone fractures among hemodialysis patients in the Dialysis Outcomes and Practice Patterns Study. Kidney Int.

[2] Kim SM, Long J, Montez-Rath M (2016). Hip fracture in patients with non-dialysis-requiring chronic kidney disease. J Bone Miner Res.

[3] Naylor KL, McArthur E, Leslie WD (2014). The three-year incidence of fracture in chronic kidney disease. Kidney Int.

[4] Tentori F, McCullough K, Kilpatrick RD (2014). High rates of death and hospitalization follow bone fracture among hemodialysis patients. Kidney Int.

[5] Vangala C, Niu J, Montez-Rath ME (2020). Hip fracture risk among hemodialysis-dependent patients prescribed opioids and gabapentinoids. J Am Soc Nephrol.

[6] Ishida JH, McCulloch CE, Steinman MA (2018). Opioid analgesics and adverse outcomes among hemodialysis patients. Clin J Am Soc Nephrol.

[7] Cohen SD, Patel SS, Khetpal P (2007). Pain, sleep disturbance, and quality of life in patients with chronic kidney disease. Clin J Am Soc Nephrol.

[8] Pham PCT, Dewar K, Hashmi S (2010). Pain prevalence in patients with chronic kidney disease. Clin Nephrol.

[9] Zhuo M, Triantafylidis LK, Li J, Paik JM (2021). Opioid use in the nondialysis chronic kidney disease population. Semin Nephrol.

[10] Novick TK, Surapaneni A, Shin JI (2018). Prevalence of opioid, gabapentinoid, and NSAID use in patients with CKD. Clin J Am Soc Nephrol.

[11] Brkovic T, Burilovic E, Puljak L (2016). Prevalence and severity of pain in adult end-stage renal disease patients on chronic intermittent hemodialysis: a systematic review. Patient Prefer Adherence.

[12] Han Y, Balkrishnan R, Hirth RA (2020). Assessment of prescription analgesic use in older adults with and without chronic kidney disease and outcomes. JAMA Netw Open.

[13] Kimmel PL, Fwu CW, Abbott KC (2017). Opioid prescription, morbidity, and mortality in United States dialysis patients. J Am Soc Nephrol.

[14] Ping F, Wang Y, Wang J (2017). Opioids increase hip fracture risk: a meta-analysis. J Bone Miner Metab.

[15] Teng Z, Zhu Y, Wu F (2015). Opioids contribute to fracture risk: a meta-analysis of 8 cohort studies. PLoS ONE.

[16] Yue Q, Ma Y, Teng Y (2020). An updated analysis of opioids increasing the risk of fractures. PLoS ONE.

[17] Saunders KW, Dunn KM, Merrill JO (2010). Relationship of opioid use and dosage levels to fractures in older chronic pain patients. J Gen Intern Med.

[18] Yoshikawa A, Ramirez G, Smith ML (2020). Opioid use and the risk of falls, fall injuries and fractures among older adults: a systematic review and meta-analysis. J Gerontol Ser A Biol Sci Med Sci.

[19] Carvalho AL, Brooks DJ, Barlow D (2022). Sustained morphine delivery suppresses bone formation and alters metabolic and circulating miRNA profiles in male C57BL/6J mice. J Bone Miner Res.

[20] Pérez-Castrillón JL, Olmos JM, Gómez JJ (2000). Expression of opioid receptors in osteoblast-like MG-63 cells, and effects of different opioid agonists on alkaline phosphatase and osteocalcin secretion by these cells. Neuroendocrinology.

[21] Elhassan AM, Lindgren JU, Hultenby K (1998). Methionine-Enephalin in bone and Joint Tissues. J Bone Miner Res.

[22] Wang X, Loram LC, Ramos K (2012). Morphine activates neuroinflammation in a manner parallel to endotoxin. Proc Natl Acad Sci U S A.

[23] Zhang P, Yang M, Chen C (2020). Toll-like receptor 4 (TLR4)/opioid receptor pathway crosstalk and impact on opioid analgesia, immune function, and gastrointestinal motility. Front Immunol.

[24] Coluzzi F, Caputi FF, Billeci D (2020). Safe use of opioids in chronic kidney disease and hemodialysis patients: tips and tricks for non-pain specialists. Ther Clin Risk Manag.

[25] Metzger CE, Swallow EA, Stacy AJ, Allen MR (2021). Adenine-induced chronic kidney disease induces a similar skeletal phenotype in male and female C57BL/6 mice with more severe deficits in cortical bone properties of male mice. PLoS ONE.

[26] Metzger CE, Swallow EA, Stacy AJ, Allen MR (2021). Strain-specific alterations in the skeletal response to adenine-induced chronic kidney disease are associated with differences in parathyroid hormone levels. Bone.

[27] Dempster DW, Compston JE, Drezner MK (2013). Standardized nomenclature, symbols, and units for bone histomorphometry: a 2012 update of the report of the ASBMR Histomorphometry Nomenclature Committee. J Bone Miner Res.

[28] Gotthardt F, Huber C, Thierfelder C (2017). Bone mineral density and its determinants in men with opioid dependence. J Bone Miner Metab.

[29] Grey A, Rix-Trott K, Horne A (2011). Decreased bone density in men on methadone maintenance therapy. Addiction.

[30] Kim TW, Alford DP, Malabanan A (2006). Low bone density in patients receiving methadone maintenance treatment. Drug Alcohol Depend.

[31] Kinjo M, Setoguchi S, Schneeweiss S, Solomon DH (2005). Bone mineral density in subjects using central nervous system-active medications. Am J Med.

[32] Milos G, Gallo LM, Sosic B (2011). Bone mineral density in young women on methadone substitution. Calcif Tissue Int.

[33] Ramli FF, Hashim SAS, Effendy NM (2021). Factors associated with low bone density in opioid substitution therapy patients: a systematic review. Int J Med Sci.

[34] Chou R, Turner JA, Devine EB (2015). The effectiveness and risks of long-term opioid therapy for chronic pain: A systematic review for a national institutes of health pathways to prevention workshop. Ann Intern Med.

[35] Hsu WY, Lin CL, Kao CH (2019). Association between opioid use disorder and fractures: a population-based study. Addiction.

[36] Ensrud KE, Blackwell T, Mangione CM (2003). Central nervous system active medications and risk for fractures in older women. Arch Intern Med.

[37] Buckeridge D, Huang A, Hanley J (2010). Risk of injury associated with opioid use in older adults. J Am Geriatr Soc.

[38] Alem AM, Sherrard DJ, Gillen DL (2000). Increased risk of hip fracture among patients with end-stage renal disease. Kidney Int.

[39] Pimentel A, Ureña-Torres P, Zillikens MC (2017). Fractures in patients with CKD—diagnosis, treatment, and prevention: a review by members of the European Calcified Tissue Society and the European Renal Association of Nephrology Dialysis and Transplantation. Kidney Int.

[40] Malluche HH, Mawad HW, Monier-Faugere MC (2011). Renal osteodystrophy in the first decade of the new millennium: Analysis of 630 bone biopsies in black and white patients. J Bone Miner Res.

[41] Baldock PA, Driessler F, Lin S (2012). The endogenous opioid dynorphin is required for normal bone homeostasis in mice. Neuropeptides.

[42] Kimmel PL, Phillips TM, Simmens SJ (1998). Immunologic function and survival in hemodialysis patients. Kidney Int.

[43] Miyamoto T, Carrero JJ, Stenvinkel P (2011). Inflammation as a risk factor and target for therapy in chronic kidney disease. Curr Opin Nephrol Hypertens.

[44] Grabulosa CC, Manfredi SR, Canziani ME (2018). Chronic kidney disease induces inflammation by increasing Toll-like receptor-4, cytokine and cathelicidin expression in neutrophils and monocytes. Exp Cell Res.

[45] Mattia C, Di Bussolo E, Coluzzi F (2012). Non-analgesic effects of opioids: the interaction of opioids with bone and joints. Curr Pharm Des.

