# Supplementary material for: Two Weeks of Continuous Opioid Treatment in an Adenine-Induced Mouse Model of Chronic Kidney Disease Exacerbates the Bone Inflammatory State and Increases Osteoclasts
Source: Calcif Tissue Int. 2024 Jun 10;115(2):174–84. doi: 10.1007/s00223-024-01239-8 (PMC11246326; doi:10.1007/s00223-024-01239-8)
Supplement: Supplementary file 1 — Supplementary file1 (DOCX 333 KB) [file 223_2024_1239_MOESM1_ESM.docx]

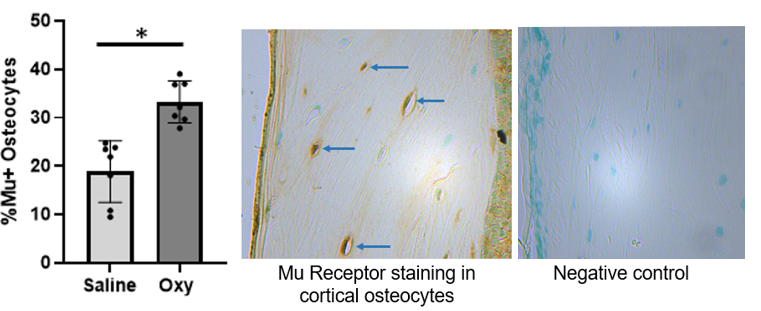


**Supplemental Figure**: Representative image of mu opioid receptor immunohistochemistry staining from mouse cortical bone (left) and a negative control image from mouse cortical bone (right).
